# Codon-Optimized NADH Oxidase Gene Expression and Gene Fusion with Glycerol Dehydrogenase for Bienzyme System with Cofactor Regeneration

**DOI:** 10.1371/journal.pone.0128412

**Published:** 2015-06-26

**Authors:** Baishan Fang, Wei Jiang, Qiang Zhou, Shizhen Wang

**Affiliations:** 1 Department of Chemical and Biochemical Engineering, College of Chemistry and Chemical Engineering, Xiamen University, Xiamen, 361005, China; 2 The Key Lab for Synthetic Biotechnology of Xiamen City, Xiamen University, Xiamen, 361005, China; 3 The Key Laboratory for Chemical Biology of Fujian Province, Xiamen University, Xiamen, Fujian, 361005, China; La Trobe University, AUSTRALIA

## Abstract

NADH oxidases (NOXs) play an important role in maintaining balance of NAD^+^/NADH by catalyzing cofactors regeneration. The expression of *nox* gene from *Lactobacillus brevis* in *Escherichia coli *BL21 (BL21 (DE3)) was studied. Two strategies, the high AT-content in the region adjacent to the initiation codon and codon usage of the whole gene sequence consistent with the host, obtained the NOX activity of 59.9 U/mg and 73.3 U/mg (crude enzyme), with enhanced expression level of 2.0 and 2.5-folds, respectively. Purified NOX activity was 213.8 U/mg. Gene fusion of glycerol dehydrogenase (GDH) and NOX formed bifuctional multi-enzymes for bioconversion of glycerol coupled with coenzyme regeneration. Kinetic parameters of the GDH-NOX for each substrate, glycerol and NADH, were calculated as *V*_max(Glycerol)_ 20 μM/min, *K*_m(Glycerol) _19.4 mM, *V*_max (NADH)_ 12.5 μM/min and *K*_m (NADH)_ 51.3 μM, respectively, which indicated the potential application of GDH-NOX for quick glycerol analysis and dioxyacetone biosynthesis.

## Introduction

NADH oxidases (NOX) catalyzes the oxidation of NADH to yield NAD^+^ and H_2_O or H_2_O_2_. NOXs play a key role in maintaining the balance of NAD^+^/NADH by regenerating coenzyme [1–5]. There are two kinds of NOXs, corresponding to H_2_O_2_-forming (NOX-1) and H_2_O-forming (NOX-2), respectively [6]. NOX-1 catalyzes the two-electron reduction of O_2_ by NADH, while NOX-2 catalyzes the four-electron reduction of O_2_ by NADH [6]. There are low homology of deduced amino acid sequence between the NOX-1 and NOX-2 [7, 8]. NOX-2 is an integrant enzyme for the NAD^+^ regeneration during aerobic mannitol metabolism, acts an important role in aerobic energy metabolism in O_2_-tolerant *Streptococcus mutans* and maintaining the balance of NAD^+^/NADH, while the NOX-1 contributes negligibly [3].

Species specific codon usage change is often considered one of the foremost causes impacting protein expression levels [9, 10]. Variations in codon usage between species is key factor for the influence of recombinant protein expression levels. Codon optimization influences on the speed of translation, in turn, changes the structure and function of proteins, and the efficiency of the protein refolding recovered from inclusions [11–13]. Codon-optimized genes strategy has a significant impact on the industrial enzyme production and different codon optimization methods have served in the past ten years [9, 14–21].

**Fig 1:**
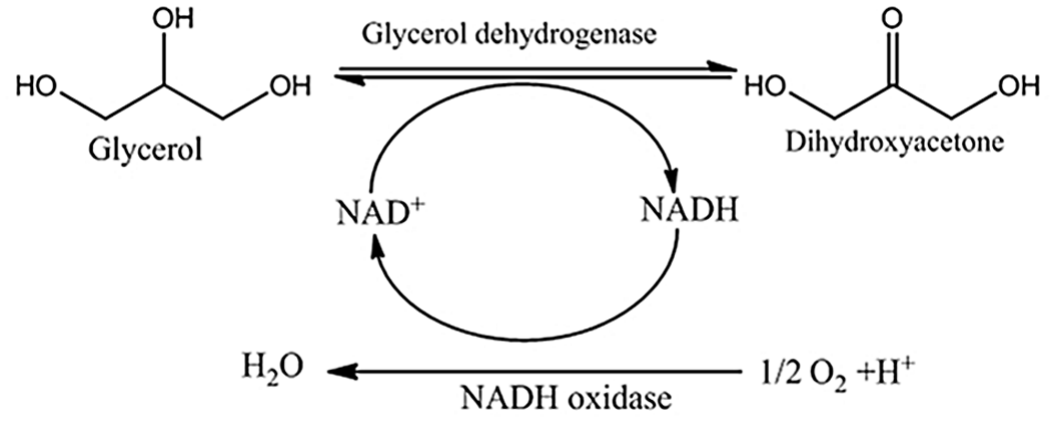
Bioconversion of glycerol by fused glycerol dehydrogenase and NADH oxidase coupled with NADH regeneration.

Glycerol dehydrogenases (GDHs) play crucial roles in the pathway of glycerol metabolism for the production of dioxyacetone (DHA) and 1, 3-propanediol (1, 3-PD). GDHs are also widely used in medical diagnosis and glycerol concentration analysis in fermentation process [22, 23]. Coupling with NADH oxidases, glycerol dehydrogenase catalyzes glycerol to dioxyacetone with NAD^+^ regeneration, which overcome the disadvantage of expensive consumption of NAD^+^ (Fig 1). Based on end-to-end fusion technique, many fusion enzymes have been developed [24, 25], which obtained enhanced catalysis efficiency.

Firstly, comparative research of the two codon optimization strategies were used to improve the expression level of the NOX by optimizing the gene nox encoding NOX from *Lactobacillus brevis* ATCC 367 (*L*. *brevis* ATCC 367). First codon optimization strategy improved the AT content of 2–6 codons downstream of the gene initiation codon and the second codon optimization strategy rearranged NOX coding sequence to keep the codon usage frequency consistent with the *E*. *coli* BL21 (DE3) codon usage frequency. To our knowledge, this is the first report to improve the NOX expression by codon optimization strategies. Secondly, gene fusion of GDH-NOX bienzyme complex by splicing overlap extension PCR (SOE-PCR) was carried out. Kinetic parameters of GDH-NOX for each substrate, glycerol and NADH were investigated. Bioconversion of glycerol into dioxyacetone coupled with coenzyme regeneration has a promising prospect of application for glycerol analysis and DHA production.

## Material and Methods

### Bacterial strains, plasmids, and reagents

The strain *L*. *brevis* ATCC 367 was obtained from the Institute of Microbiology of the Chinese Academy of Sciences. The strain *K*. *pneumoniae* DSM2026 was obtained from Doctor An-Ping Zeng (Hamburg University of Technology). *E*. *coli* DH5α was used as host strains for cloning. BL21 (DE3) was used as host strains for expression. Plasmid pET-32a(+) was employed as an expression vector.

All enzymes, such as restriction endonucleases, T4 DNA ligase and Ex *Taq* DNA, were recruited from TaKaRa Co., Ltd. (Dalian, China). PrimeSTAR HS DNA Polymerase, Ligation solution I, Agarose Gel DNA Purification Kit Ver 2.0, Mutan BEST Kit, Agarose Gel DNA Fragment Recovery Kit Ver.2.0 and pMD18-T were obtained from TaKaRa Co., Ltd. (Dalian, China). GeneRuler Ladder Mix was purchased from MBI Co. All other chemicals used were analytically graded and were purchased from either Sigma China or Omiga China.

### Gene cloning and recombinant plasmid construction

The *nox* gene (Gene ID: CP000416) was cloned by the polymerase reaction (PCR) in the following three steps: (i) an initial denaturation step at 95°C for 5 min; (ii) 30 cycles of amplification (denaturation at 95°C for 1 min, annealing at 63°C for 50s and extension at 72°C for 2 min); and (iii) a final extension at 72°C for 10 min, with the forward and reverse primers (*nox*P1 and *nox*P2, Table 1) containing *Bam*HI and *Xho*I sites (underlined), respectively. The purified PCR products by gel purification were treated with *Bam*HI and *Xho*I before reassembled into expression vector pET-32a(+), generating pET-32a-*nox*. Then the recombinant plasmids were transformed into competent *E*. *coli* DH5α and cultivated at 37°C. The recombinant plasmids were sequenced and the positive recombinant plasmids were transformed into BL21 (DE3) for protein expression.

**Table 1:** Oligonucleotide primers used in this study.

Primers	5' to 3'
*nox*P1	CGGGATCCATGAAAGTCACAGTTGTTGG
*nox*P2	CCGCTCGAGAGCGTTAACTGATTGGG
PT01	TAACTGTTGTAGGTTGTACACATGCC
PT02	TAACAGTTGTTGGTTGTACACATGCC
PT03	TAACAGTTGTAGGTTGTACACATGCC
PT04	TAACTGTAGTAGGTTGTACACATGCC
PT05	TAACTGTAGTTGGTTGTACACATGCC
PT06	TTACAGTAGTTGGTTGTACACATGCC
PT07	TTACTGTAGTAGGTTGTACACATGCC
PT08	TAACAGTAGTTGGTTGTACACATGCC
P	CTTTCATGGATCCGATATCAGCCATG
P1	CGGGATCCATGCTAAAAGTTATTCAATCTCC
P2	CCAACAACTGTGACTTTCATACGCGCCAGCCACTGCTGG
P3	CCAGCAGTGGCTGGCGCGTATGAAAGTCACAGTTGTTGG
P4	CCGCTCGAGAGCGTTAACTGATTGGG

### Expression and purification of the NOX

The transformant was selected from a single colony, and grown overnight at 37°C in LB medium (ampicillin 100 μg/mL). Subsequently, the culture was inoculated into fresh LB medium (1:100 dilution, containing 100 μg/mL ampicillin), at 37°C. At an optical density (OD600) of 0.5–0.6, IPTG was added to a final concentration of 1.0 Mm, and the mixture was incubated at 37°C. Then, cells were harvested for enzyme assay. Cell free extract was obtained by the follow steps: the induced restructuring cell was centrifuged 10 min at 1°C, 8000 g/min, before being disrupted using a French cell press at 20,000 psi cell pressure; the cell lysate was centrifuged at 12,000 rpm for 10 min at 4°C, and the supernatant was collected. Then his-tagged enzymes were purified by using HisTrap HP column.

### Enzyme assay and biochemical characterization of the NOX

The NOX activity was determined at 37°C by monitoring the oxidation of NADH at 340 nm as described previously [26], with modification. The principle is based on that NOX can catalyze NADH to H_2_O, and the NADH which can be detected by spectrophotometer at 340 nm. 1 ml of the reaction system was consisted of of GDH, 0.2 mM NADH, 0.035 mol/L potassium phosphate buffers. The optimum pH of the NOX was determined at 37°C in different buffers with pH ranging from 3 to 10. The optimum operation time was determined by comparison of NOX activity with different operation time (6, 8, 10 and 12 min). The NOX activity with different inducing time (1, 2, 4, 6, 8h) was compared in order to determine the optimum inducing time.

Kinetic parameters, *V*_*m*_ and *K*_*m*_ of the purified NOX, were determined by measuring the enzyme activity with the substrate concentration of 20, 40, 80, 100, 200 and 400 μM, at the optimum pH and temperature. One unit of NOX activity is defined as the amount of enzyme needed to catalyze the reduction of 1 μmol of NADH per min under standard conditions. The data of the activity of NOX was detected under the standard method.

### Gene design, synthesis and expression vector construction

#### Codon optimization by site directed mutagenesis

It had been showed that the open reading frame (ORF) initiation codon downstream area (DB) can effectively affect the translation efficiency of prokaryote and optimizing the content of the AT in this region can effectively improve the level of gene expression [27]. This method would be improving the expression efficiency of the NOX in host cells. In this paper, the synonymous codon mutation was implemented during the third and sixth codon of the *nox*, and the content of the AT increase from 66.7% (the wild type) to 73.3% (the mutant strain). Using the pET-32a-*nox* as template, site directed mutagenesis mutants were obtained by PCR using 8 kinds of mutation primers (PT01-08, Table 1) and 1 kind of downstream primers P (Table 1) under the following conditions: 1 cycle at 94°C for 1 min; 30 cycles at 98°C for 10 s, 59°C for 15 s and 72°C for 8 min; and 1 final additional cycle at 72°C for 10 min. The resulting PCR product was gel-purified, then connected through ligation solution I in vitro and transformed into the BL21 (DE3). The recombinant plasmids were verified by DNA sequencing, and successfully introduced desired mutations were designated as T01-08.

#### Codon optimization and redesign of the NOX

Based on the codon usage of *E*. *coli* (http://www.kazusa.or.jp/codon), the full sequence of the *nox* was redesigned without changing the amino acid sequence in order to improve the expression level of the NOX in BL21 (DE3). Codon usage of wild type and optimized genes was showed in Table 2. According to the codon usage frequency, put the give priority codes in front of the gene, namely as the length of the gene codon usage frequency from high to low, that it is helpful to improve the efficiency of translation [28]. The optimized gene (Nox-opt) was synthesized by Sangon Biotech and cloned into the pET-32a(+), generating opt-*nox*.

**Table 2:** Codon usage of wild type and optimized genes.

		Codon usage of *E*. *coli B*		Wild type		Optimized	
AA	Codon	Number	Frequency	Number	Frequency	Number	Frequency
Gly	GGA	3	0.1	3	0.11	3	0.1
	GGT	9	0.3	15	0.54	9	0.3
	GGC	11	0.41	6	0.21	11	0.41
	GGG	5	0.18	4	0.14	5	0.18
Ala	GCA	10	0.21	11	0.23	10	0.21
	GCT	7	0.15	15	0.32	7	0.15
	GCC	13	0.28	13	0.28	13	0.28
	GCG	17	0.36	8	0.17	17	0.36
Val	GTA	5	0.14	1	0.03	5	0.14
	GTT	9	0.25	16	0.44	9	0.25
	GTC	6	0.18	13	0.36	6	0.18
	GTG	16	0.43	6	0.17	16	0.43
Leu	TTA	5	0.14	13	0.34	5	0.14
	TTG	6	0.15	4	0.11	6	0.15
	CTA	1	0.03	7	0.18	0	0.00
	CTT	4	0.11	3	0.08	4	0.11
	CTC	5	0.12	7	0.18	5	0.12
	CTG	17	0.45	4	0.11	18	0.48
Ile	ATA	2	0.07	0	0.00	0	0.00
	ATT	14	0.48	18	0.64	16	0.55
	ATC	12	0.44	10	0.36	12	0.44
Met	ATG	13	1.00	13	1.00	13	1.00
Phe	TTT	8	0.61	10	0.77	8	0.61
	TTC	5	0.39	3	0.23	5	0.39
Tyr	TAT	12	0.69	7	0.41	12	0.69
	TAC	5	0.31	10	0.59	5	0.31
Trp	TGG	2	1.00	2	1.00	2	1.00
Ser	TCA	2	0.11	9	0.35	2	0.11
	TCT	4	0.15	8	0.31	4	0.15
	TCC	4	0.14	1	0.04	4	0.14
	TCG	5	0.2	1	0.04	5	0.2
	AGT	4	0.16	4	0.15	4	0.16
	AGC	7	0.25	3	0.11	7	0.25
Pro	CCA	4	0.18	6	0.3	4	0.18
	CCT	3	0.14	4	0.2	3	0.14
	CCC	1	0.06	5	0.25	0	0.00
	CCG	12	0.61	5	0.25	13	0.67
Thr	ACA	4	0.11	6	0.18	4	0.11
	ACT	5	0.14	11	0.32	5	0.14
	ACC	16	0.47	9	0.26	16	0.47
	ACG	9	0.27	8	0.24	9	0.27
Cys	TGT	2	0.42	2	0.42	2	0.42
	TGC	3	0.58	3	0.58	3	0.58
Asn	AAT	13	0.57	8	0.35	13	0.57
	AAC	10	0.43	15	0.65	10	0.43
Gln	CAA	7	0.35	19	0.9	7	0.35
	CAG	14	0.65	2	0.1	14	0.65
Lys	AAA	17	0.77	12	0.55	17	0.77
	AAG	5	0.23	10	0.45	5	0.23
His	CAT	6	0.56	5	0.5	6	0.56
	CAC	4	0.44	5	0.5	4	0.44
Arg	CGA	1	0.05	1	0.08	0	0.00
	CGT	4	0.35	3	0.25	5	0.4
	CGC	5	0.4	2	0.17	5	0.4
	CGG	1	0.11	5	0.42	2	0.2
	AGA	1	0.05	1	0.08	0	0.00
	AGG	0	0.04	0	0.00	0	0.00
Asp	GAT	23	0.66	25	0.71	23	0.66
	GAC	12	0.34	10	0.29	12	0.34
Glu	GAA	12	0.62	17	0.85	12	0.62
	GAG	8	0.38	3	0.15	8	0.38

#### Enzyme assay of the T01-08 and the opt-nox

Enzyme activity can be invoked as a measure index of recombinant protein expression level [29]. So, the change in amount of protein expression could be determined by detected the activity of the T01-08 and the opt-*nox*. After incubated by IPTG, his-tagged enzymes were purified by using HisTrap HP column.

#### Fusion enzyme gene construction

The *gdh* gene, from *K*. *pneumoniae* DSM2026, was cloned by the PCR in the following three steps: (i) an initial denaturation step at 95°C for 1 min; (ii) 30 cycles of amplification (denaturation at 95°C for 30s, annealing at 60°C for 30s and extension at 72°C for 2 min); and (iii) a final extension at 72°C for 10 min, with the forward and reverse primers (P1 and P2, Table 1) containing *Bam*HI sites in the forward primer (underlined). The *nox* gene was obtained by the same method, with the forward and reverse primers (P3 and P4, Table 1) containing *Xho*I sites in the reverse primer (underlined). The PCR products were purified, generating the template of the overlapping PCR (SOE-PCR). The fusion gene, *gdh*-*nox*, was obtained by SOE-PCR using P1 and P4 primers with the following steps: 1 cycle at 95°C for 1 min; 30 cycles at 98°C for 10 s, 60°C for 15 s and 72°C for 3 min; and 1 final additional cycle at 72°C for 10 min. The purified PCR products were addressed with *Bam*HI and *Xho*I before reassembled into expression vector pET-32a (+), generating pET-32a-*gdh*-*nox*. The recombinant plasmids were sequenced and the positive recombinant plasmids were transformed into BL21 (DE3) for protein expression.

#### Enzyme assay and biochemical characterization of the GDH-NOX

The enzyme measurement system of the NOX was consisted of 0.2 mM NADH, 100 μL buffers with different pH and a moderate amount of crude enzyme. The activity of the GDH was determined by adding moderate crude enzyme under the following conditions: 30 mM (NH_4_) _2_SO_4_, 0.2 M glycerol, 2 mM NAD^+^ and 0.1 M buffer with different pH [30]. To determine optimal temperatures for the NOX and GDH activities, the fusion enzyme was assayed at the gradient temperatures of 25–55°C in the above reaction system. The fusion enzyme solution for the reaction was adjusted to pH 5.0–12.0 to detect optimal pH for the NOX and GDH. The *V*_*m*_ and *K*_*m*_ toward glycerol and NADH of the GDH-NOX were determined by measuring the enzyme activity with the glycerol substrate (concentration of 0.01, 0.125, 0.014, 0.025 and 0.05 M) and the NADH substrate (concentration of 20, 40, 60, 100 and 200 μM) respectively, at the optimum conditions.

#### DHA biosynthesis

After activation, the strains were inoculated in 200 mL LB medium (1:100 dilution, containing 100 μg/mL ampicillin), and grown at 37°C for 2 h. At an OD600 of 0.5–0.6, IPTG was added to a final concentration of 1.0 mM, and the mixture was incubated at 37°C for 4h. Then, cells were harvested for catalytic glycerol producing DHA. According to per 1g thallus 10 mL buffers, the cell free extract was obtained. The catalytic reaction experiments, which were used to explore the productivity of pET-32-*nox*, pET-32-*gdh* and pET-32-*gdh*-*nox* in DHA production, were implemented with 10 g/L glycerol as substrate.

Glycerol concentration was quantified by a colorimetric method at 450nm as described previously [31], and the principle is based on that acidic periodate oxidation sugar alcohol produce formaldehyde, generated under Nash reagent in yellow compounds, the compounds have the largest under 450 nm absorption peak, and the depth of the color and glycerin concentration is proportional, the glycerin concentration can be quantitatively by colorimetric method. Using the ability of reducing, the content of DHA can be determined by colorimetric method which was described in the paper [32], the principle is that DHA has a reducing activity, under the condition of boiling, can react with phosphor molybdate reagent, generate molybdenum blue, make the solution is blue, the color depth is proportional to the concentration of DHA.

## Results

### Gene cloning, expression, purification and NOX characterization

**Fig 2:**
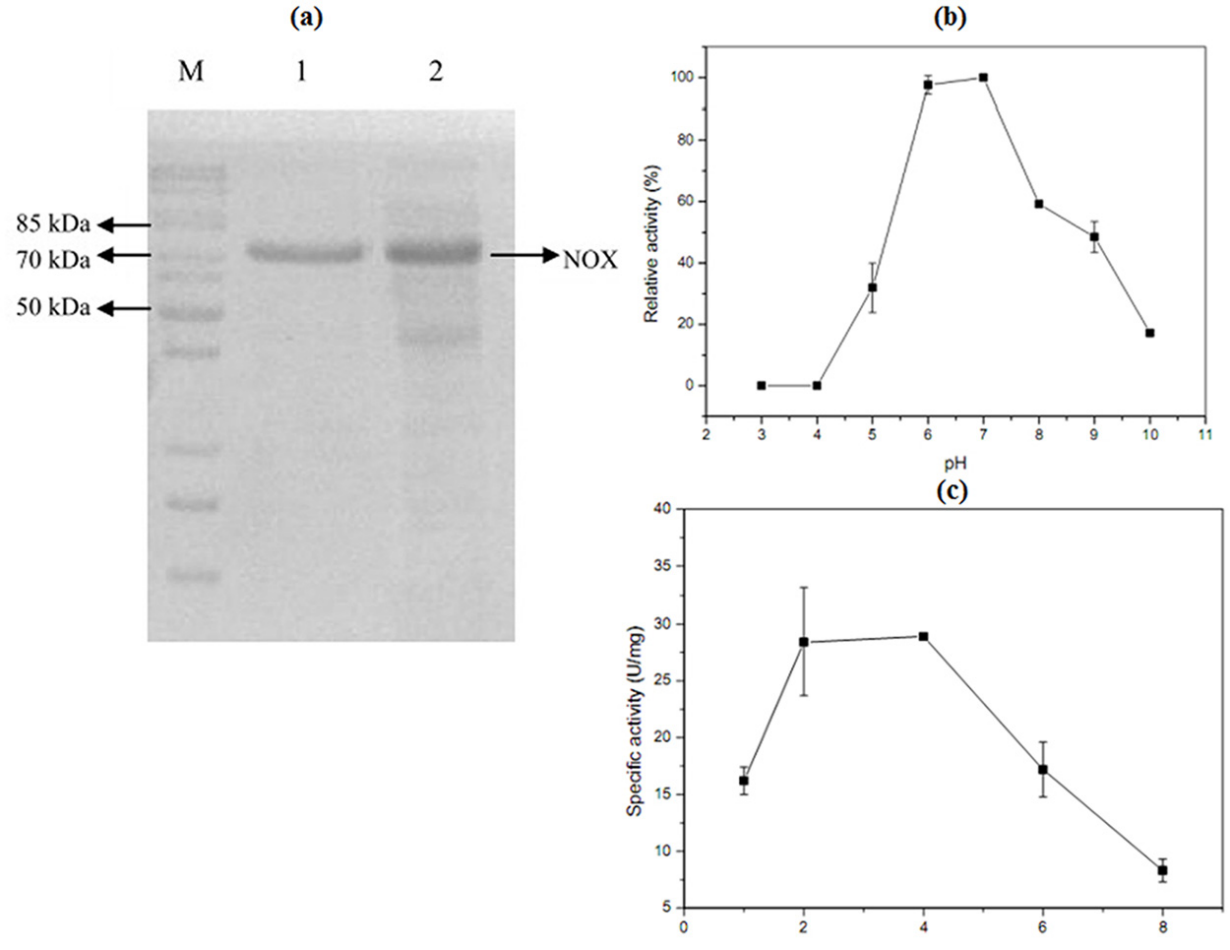
Characterization of the NOX and SDS-PAGE analysis of the purified NOX. (a) 10% SDS-PAGE analysis of the purification NOX. Lane M: protein marker; Lane 1: purified NOX with His-tag; Lane 2: recombinant bacterium (harboring pET-32a-*nox*) induced by IPTG. (b) Optimal pH for NOX. (c) The optimal inducing time of NOX.

**Fig 3:**
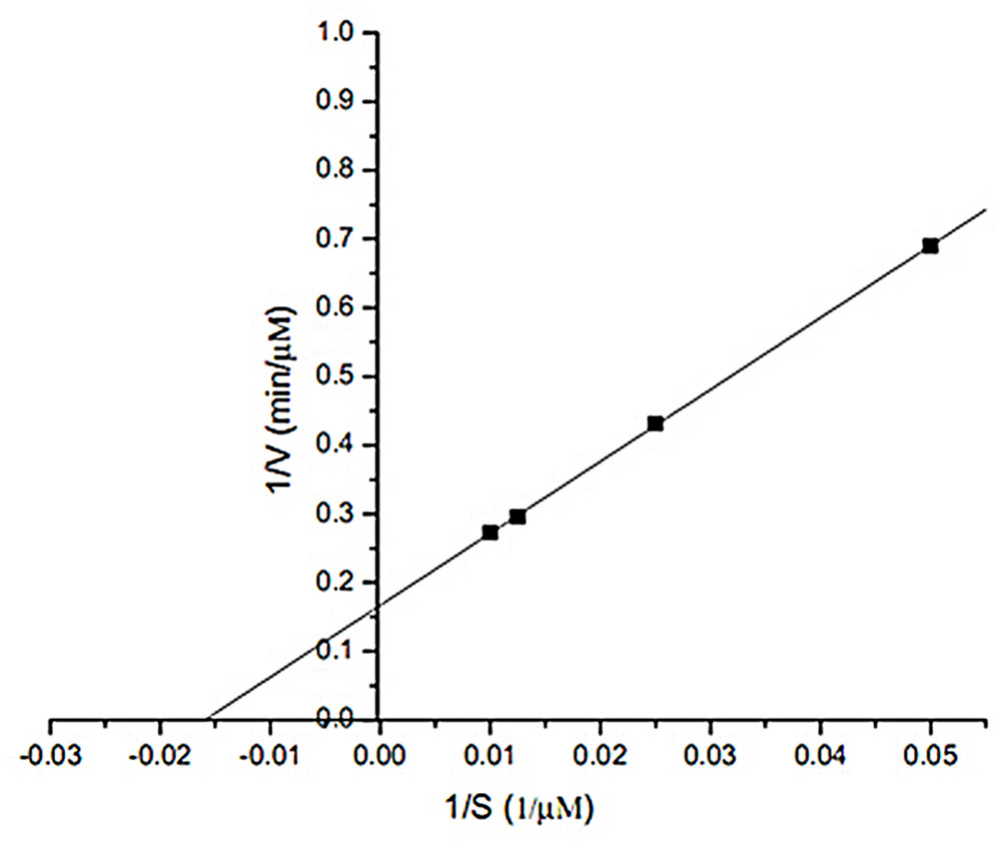
Double reciprocal plot for *K*_m_ and *V*_max_ of NOX. Experiment condition: NADH concentration (20, 40, 60, 100, 200 μM), pH 7.0, 37°C.

The *nox* gene (1353 bp) encoding a polypeptide of 457 amino acids was obtained with a deduced molecular mass of 48.9 kDa. A single band of the purified NOX was showed in SDS—PAGE picture (Fig 2a). Optimal pH was 7.0 (Fig 2b). NOX demonstrated the highest specific activity of 28.9 U/mg after 4 h induction (Fig 2c). Kinetic parameters, *K*_m_ and *V*_max_, were calculated as 62.6 μM and 5.99 μM/min, respectively (Fig 3).

### Gene design, synthesis and expression vector construction

**Fig 4:**
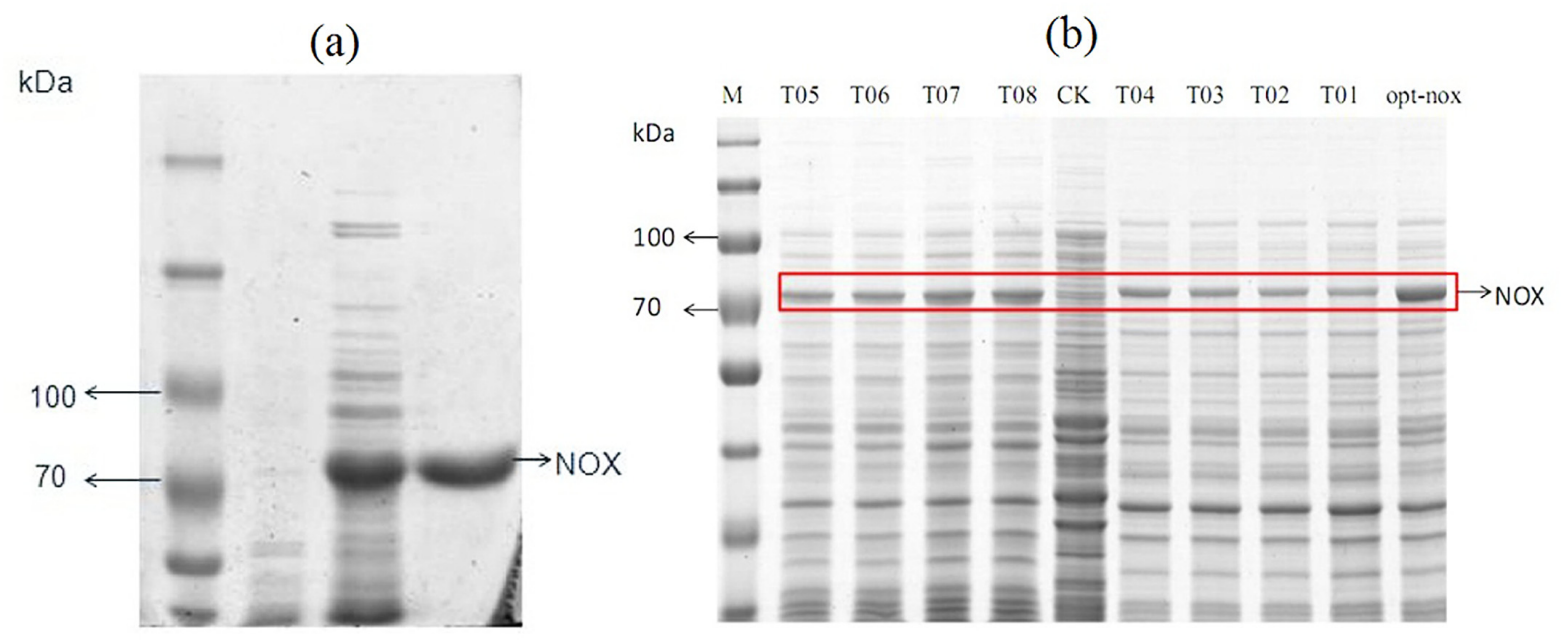
Alignment of wild type nucleotide sequence (*nox*) with the optimized sequence (Nox-opt). Identical residues were highlighted in black background.

The first optimization strategy was based on improving the AT content of 2–6 codons downstream of the initiation codon. The mutants (T01-08) were obtained by site directed mutagenesis and expressed in BL21 (DE3). Comparison of mutation sites with the wild-type was showed in Table 3. For the second optimization strategy, gene sequence was rearranged for keep the codon usage frequency consistent with the *E*. *coli* BL21 (DE3) codon usage frequency. The optimized NOX gene (Nox-opt) was synthesized according to the codon optimization scheme and its sequence comparison with wild-type sequence was showed in Fig 4. Codon adaptation index (CAI) of the optimized sequence of the Nox-opt was 0.53, which was calculated by Codon-Adaptation Tool [33], was higher than that of the wild type (0.39), suggesting the optimized Nox-opt would be better in the expression in the host cell.

**Table 3:** Sequences of the third to sixth codons and the Nox activities.

Name	AT-content in the third to sixth codons (%)	Sequence of the third to sixth codons	Nox specific activity (U/mg)
wild	58.3	GTCACAGTTGTT	28.9 ± 0
T01	66.7	GTAACTGTTGTA	27.9 ± 1.6
T02	66.7	GTAACAGTTGTT	38.5 ± 0.7
T03	66.7	GTAACAGTTGTA	40.2 ± 1.2
T04	66.7	GTAACTGTAGTT	41.3 ± 3.4
T05	66.7	GTAACTGTAGTA	47.2 ± 0.5
T06	66.7	GTTACTGTAGTA	49.1 ± 8.1
T07	66.7	GTAACAGTAGTT	54.5 ± 0.4
T08	66.7	GTTACAGTAGTT	59.9 ± 4.9

Note: Mutation sites was underlined.

### Enzyme activity of the T01-08 and the opt-nox

**Fig 5:**
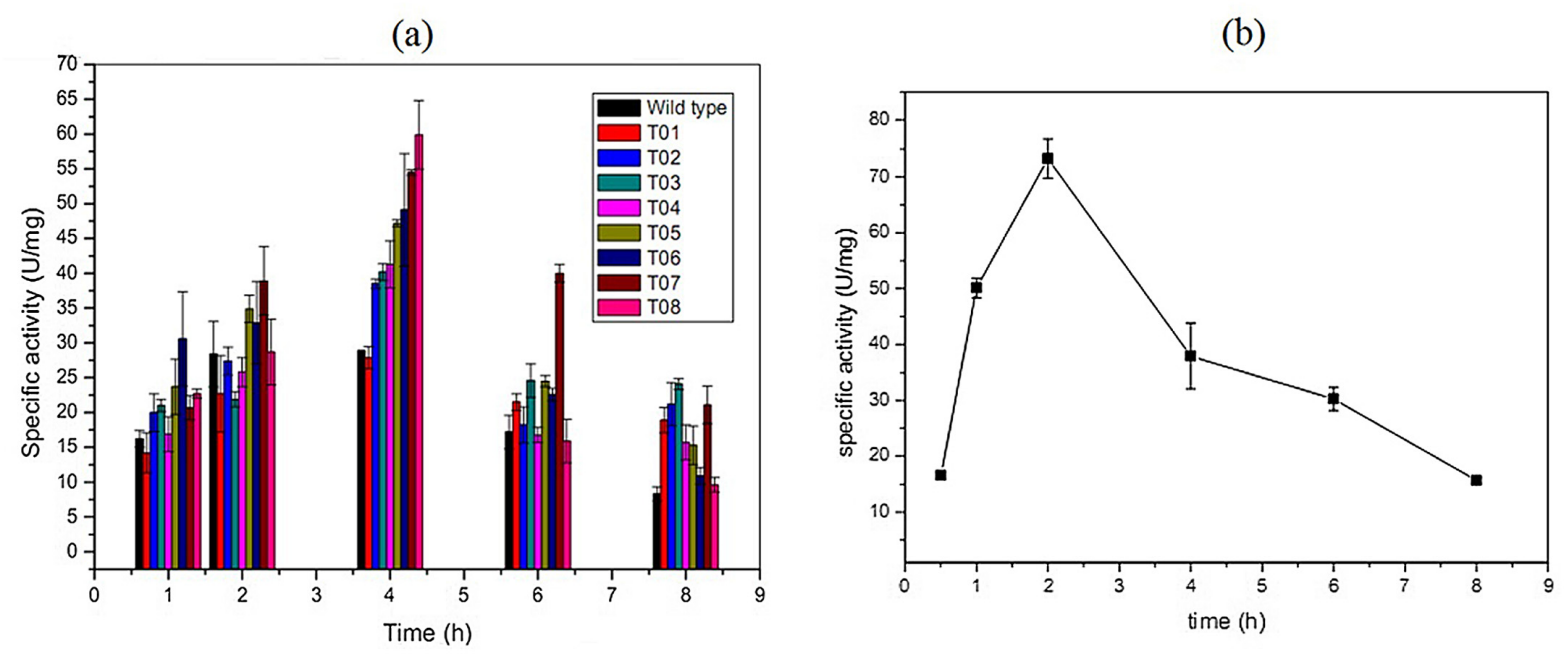
Optimal induce time of the T01-08, opt-*nox* and the wild type. (a)Comparison of the specific activity of the T01-08 and the wild type with different induced time (1–8h). (b) Optimal inducing time of the opt-*nox*.

Optimum inducing time of the mutant strains T01-08 was determined by comparison of T01-08 activities with different inducing time (1, 2, 4, 6, 8h). It could be noted that the T01-08 exhibited the highest activities after 4 h induction (Fig 5a), which was showed in Table 3. Increasing AT content in mutation area, the specific activity improved. NOX activity in cell extract of the mutants was 59.9 U/mg, which was 2.0-folds of the wild type (28.9 U/mg).

**Fig 6:**
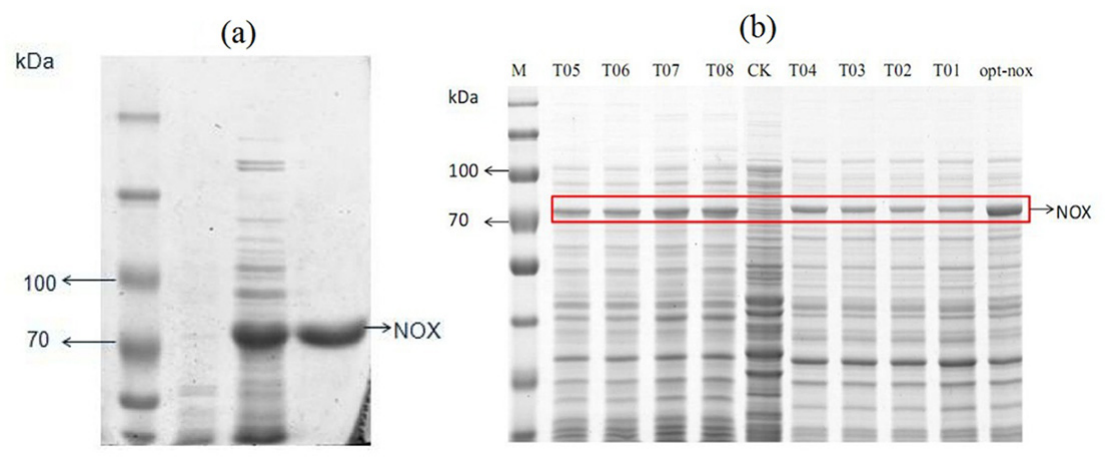
Purification of NOXs and characterization of the optimized opt-*nox* expression levels by SDS-PAGE. (a) 10% SDS-PAGE analysis of the purification opt-*nox*. M: protein marker; Lane 1: bacterium (harboring pET-32a) induced by IPTG; Lane 2: recombinant bacterium (harboring pET-32a-opt-*nox*) induced by IPTG; Lane 3: purified opt-*nox* with His-tag. (b) Expression levels of optimized NOXs in BL21 (DE3) were analyzed by 10% SDS-PAGE. Lane M: molecular mass markers. Lane CK: BL21 (DE3) without *nox* gene. Lane T01 to T08 are different mutations. Lane opt-*nox*: the whole optimization of the *nox* sequence.

Opt-*nox* activities with different inducing time (1, 2, 4, 6, 8 h) were compared in order to determine the optimum inducing time (Fig 5b). NOX activity in cell extract of the opt-*nox* was 73.3 U/mg after 2 h induction, and it was 2.5-folds of the wild type. Then his-tagged opt-*nox* was purified by using HisTrap HP column (Table 4) and the specific activity of the purified enzyme was 213.8 U/mg. Purified opt-*nox* showed a single band in 10% SDS-PAGE (Fig 6a). SDS-PAGE was used to analyze the quantity of NOX in order to exhibit the improvement of the protein expression level (Fig 6b), which demonstrated that protein expression amount and specific activity presented certain positive correlation.

**Table 4:** Specific activities of the opt-*nox* in the process of purification.

Purificationstep	Activity (U/mL)	Protein concentration(mg/mL)	Specific activity(U/mg)	Purificationfold
Cell extract	76.2	1.04	73.3	1
Histrap HP	38.5	0.18	213.8	2.9

### Fusion of the GDH-NOX

**Fig 7:**
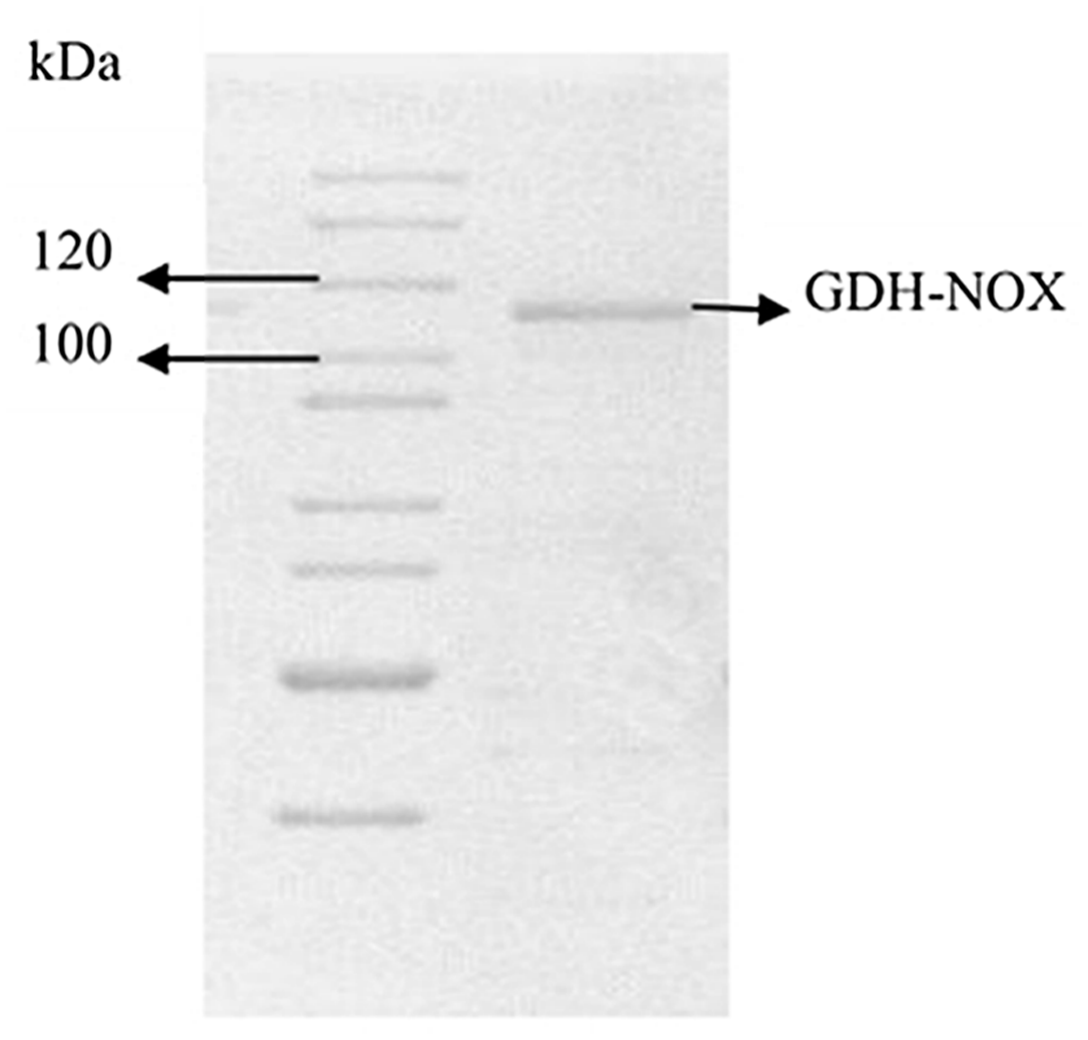
10% SDS-PAGE analysis of the purification fused GDH-NOX. Line 1: protein marker; Lane 2: purified GDH-NOX with His-tag.

**Fig 8:**
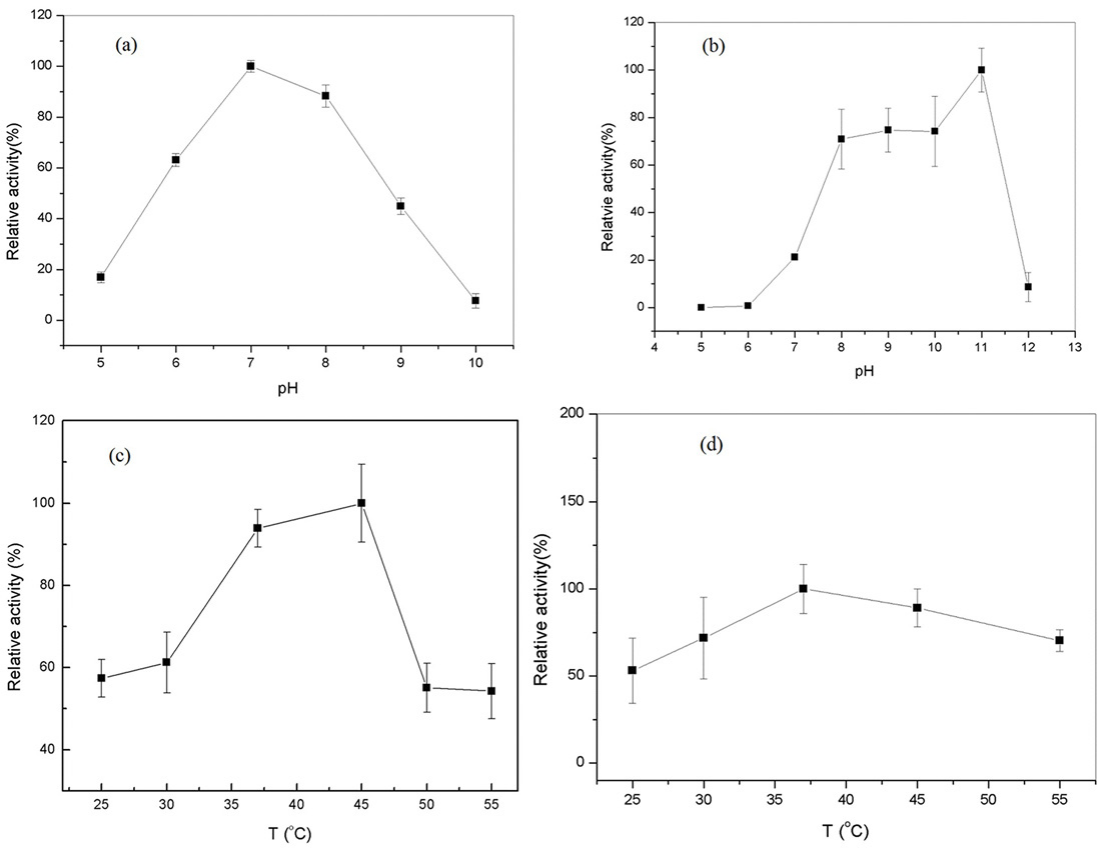
Effects of pH and temperature on the activities of the GDH-NOX. (a) optimal pH of NOX in GDH-NOX complex. (b) The optimal pH of GDH in GDH-NOX complex. (c) Optimal temperature for the GDH in the GDH-NOX complex. (d) Optimal temperature for the NOX in the GDH-NOX complex. NOX activity was tested with 0.2 mM NADH, 100 μL buffers with different pH. GDH activity was determined under the following conditions: 30 mM (NH_4_) _2_SO_4_, 0.2 M glycerol, 2 mM NAD^+^ and 0.1 M buffer with different pH.

**Fig 9:**
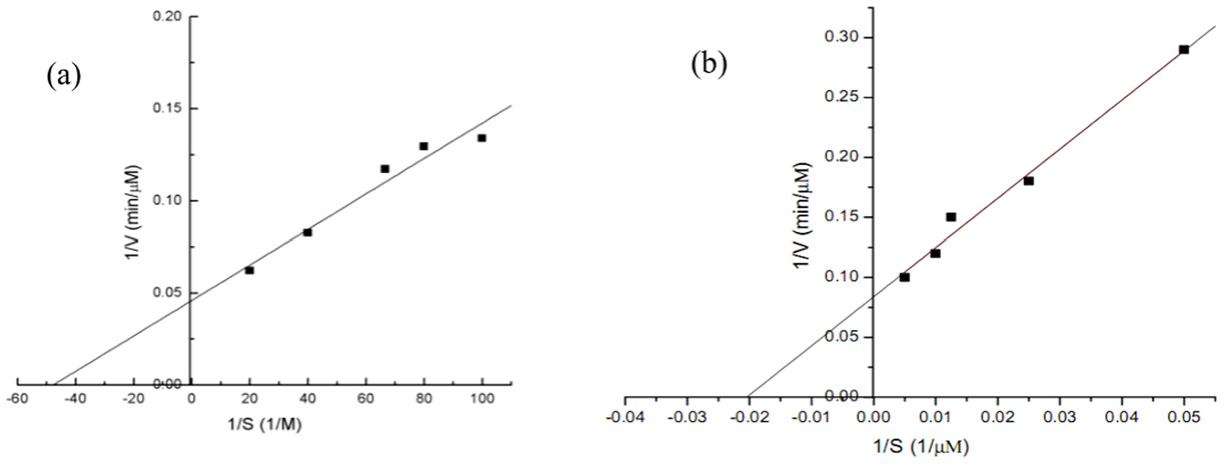
Determination of kinetic parameters of GDH-NOX. (a) Glycerol; (b) NADH. Experiment condition: glycerol concentration (0.01, 0.125, 0.014, 0.025, 0.05M), NADH concentration (20, 40, 60, 100, 200 μM). pH 7.0, 37°C.

The full-length sequence of the fusion *gdh*-*nox* gene was obtained. The enzymes were purified by using HisTrap HP column and SDS—PAGE showed a band of the purified GDH-NOX with His-tag (Fig 7). Among the bifunctional fused enzyme, the optimal pH for NOX was pH 7.0 while for the GDH was 11.0 (Fig 8a and 8b). The optimum temperature of the GDH and NOX among the GDH-NOX was 45°C and 37°C, respectively (Fig 8c and 8d). The specific activities of the GDH and NOX of the GDH-NOX were 15.1 U/mg and 15.7 U/mg, respectively. Kinetic parameters of the GDH-NOX for two substrates, glycerol and NADH, were calculated as *V*_max(Glycerol)_ 20 μM/min, *K*_m(Glycerol)_ 19.4 mM, *V*_max (NADH)_ 12.5 μM/min and *K*_m (NADH)_ 51.3 μM, respectively (Fig 9).

### Enzymatic synthesis of DHA

Bioconversion of glycerol to DHA by the cell extracts of the BL21 (DE3), pET-32-*nox*, pET-32-*gdh* and pET-32-*gdh*-*nox*, were implemented. Glycerol conversion rates were showed in Table 5, which indicated that glycerol conversion rates of the pET-32a-*gdh*-*nox* was found to be 3.5-folds and 8.6-folds of the pET-32a-*gdh* at 10 and 30min, respectively.

**Table 5:** Bioconversion of glycerol by GDH and GDH-NOX.

Time (min)	Glycerol conversion rate (%)	
	GDH	GDH-NOX
10	2.6	19.6
30	3.7	31.7

## Discussion

NOXs are key enzymes for the regulation of aerobic metabolism and regenerating NAD^+^ [3]. To reduce the cost of enzymes production, increasing NOX expression level by codon optimization is an effective strategy. Two strategies, high AT-content in the region adjacent to the initiation codon and codon usage of the whole gene sequence consistent with the host, have dramatically enhanced translation. NOX activity in the crude extract, 28.9 U/mg, was higher than those NOXs from other *Lactobacillus* strains (*L*. *brevis* DSM 20 054, 10.6 U/mg; *L*. *kefir*, 5.4 U/mg; *L*. *casei*, 0.4 U/mg; *L*. *mesenteroides*, 0.5 U/mg) [34].

Due to fast growth rate, cheap fermentation media and clear genetics, *E*. *coli* is a preferred host for recombinant proteins production [15, 16]. The 5’ coding region is the most sensitive to codon usage for expression levels and particularly critical in modulating translation initiation [16, 35–38]. NOX activity of the gene with high AT-content in the region adjacent to the initiation codon by local optimization was 59.9 U/mg in cell extract. AT-rich content in the initiation codon downstream sequence improved the efficiency of protein translation, which may be through improving the structure of the mRNA, reduce the activation energy and easy to form translation starting compounds [27]. This positional effect is due to an effect on the stability of translation complexes near the beginning of a message [39].

To raise the protein expression level, the replacement of rare gene codes for optimal codon or increase the number of rare tRNA in the host [40] are applied. Codon optimization was used to enhance the F2 domain EBA-175 expression in both *E*. *coli* and *P*. *pastoris* [41]. NOX was synthesized according to *E*. *coli* codon usage and the CAI value was improved from 0.39 to 0.53. CAI value is between 0 and 1, the larger the more codon bias, which is used to predict the level of gene in heterologous hosts. NOX activity was up to 73.3 U/mg in cell extract when the gene sequence consistent with the host, which is almost 2.5-folds of the wild type. NOX activity of the purified opt-*nox* was 213.8 U/mg, which was 1.84-folds of the *L*. *brevis* DSM 20 054 (116 U/mg) [26]. Codon bias has been taken into account for efficient protein expression, reduced the metabolic load by reduced diversity of the isoacceptor tRNA for increasing the expression of a heterologous gene in a host [21, 35, 42]. The opt-*nox* showed the highest activity after induced 2 h while the optimum inducing time for pET-32a-*nox* was 4 h, which indicated the codon optimization could also save the inducing time (Fig 5).

Biocatalysis is green process for chemical synthesis on an industrial scale [43]. GDH was reported to be inactivated by oxidation under aerobic conditions [44–46]. NAD^+^ is expensive and the generated reduced coenzyme NADH is the competitive inhibitor of NAD^+^ [23, 47]. Therefore, *in situ* recycling coenzyme can overcome those shortcomings. The GDH-NOX fusion protein was constructed by SOE-PCR. The *V*_*max*_ value of the GDH-NOX towards glycerol and NADH deceased, which may be due to the fusion protein generating space steric effect for the substrate. The C-terminal of the NOX fusion with the N-terminus of the GDH, may hinder the catalytic sites.

Bifunctional activities of GDH-NOX fusion enzyme could be enhanced by adding the peptide linkers, which separates the two enzymes at a reasonable distance. With optimized peptide linkers, the performance of the β-glucanase-xylanase fusion was enhanced [48]. The amount of glycerol can be measured in a few minutes (Table 5), suggesting the GDH-NOX bienzymes coupled with coenzyme regeneration system can be used to detect the glycerol concentration inside of red wine, human blood and on-line monitoring in fermentation process.

## Conclusion

Codon optimization strategies were developed to enhance the NADH oxidase expression in *E*. *coli*. NOX activity of the *nox* gene with high AT-content in the region adjacent to the initiation codon and the *nox* gene sequence consistent with the host were 2.0 and 2.5-folds of the wild type, respectively. The fusion GDH-NOX with coenzyme regeneration has the potential application for glycerol analysis and enzymatic production of DHA.
